# Peroneal neuropathy misdiagnosed as L5 radiculopathy: a case report

**DOI:** 10.1186/2045-709X-21-12

**Published:** 2013-04-22

**Authors:** Michael D Reife, Christopher M Coulis

**Affiliations:** 1 Private Practice, 8 Independence Drive, Marlborough, CT 06447, USA; 2 Basic and Clinical Sciences, University of Bridgeport College of Chiropractic, 75 Linden Ave, Bridgeport, CT 06604, USA; 3 Department of Veterans Affairs Connecticut Healthcare System, West Haven, CT, 06516, USA; 4 Shoreline Spine & Pain Associates, PC, 2415 Boston Post Rd., Guilford, CT, 06437, USA; 5 Clinical Sciences, University of Bridgeport College of Chiropractic, 75 Linden Ave., Bridgeport, CT, 06604, USA

**Keywords:** Peroneal neuropathy, Lumbar radiculopathy, Chiropractic

## Abstract

**Objective:**

The purpose of this case report is to describe a patient who presented with a case of peroneal neuropathy that was originally diagnosed and treated as a L5 radiculopathy.

**Clinical features:**

A 53-year old female registered nurse presented to a private chiropractic practice with complaints of left lateral leg pain. Three months earlier she underwent elective left L5 decompression surgery without relief of symptoms.

**Intervention and outcome:**

Lumbar spine MRI seven months prior to lumbar decompression surgery revealed left neural foraminal stenosis at L5-S1. The patient symptoms resolved after she stopped crossing her legs.

**Conclusion:**

This report discusses a case of undiagnosed peroneal neuropathy that underwent lumbar decompression surgery for a L5 radiculopathy. This case study demonstrates the importance of a thorough clinical examination and decision making that ensures proper patient diagnosis and management.

## Background

The most common entrapment neuropathy in the lower extremity is common peroneal mononeuropathy, accounting for approximately 15% of all mononeuropathies in adults [1]. Most injuries occur at the fibular head and can be the result of many factors including chronic low grade infection [2], varicose veins [3], schwannoma [4], nerve herniation through a fascial defect [5], giant plexiform neurofibromatosis [6], pneumatic compression [7], total knee arthroplasty, proximal tibial osteotomy [8], ganglion cysts [9], weight loss [10], associated endocrine or metabolic disorders including diabetes mellitus, alcoholism, thyrotoxicosis or Vitamin B depletion [11], high ankle sprain and leg crossing/squatting [12,13]; the most common cause of which is habitual leg crossing [14]. In this paper, we present a case of peroneal neuropathy that was originally misdiagnosed as a lumbar radiculopathy.

## Case presentation

The patient is a 53-year-old registered nurse who was referred to the author’s office in August 2003 by her primary care physician with a chief complaint of left leg pain. Her symptoms began in October 2002 after she fell off an ambulance landing on her left hip. Plain x-rays of her hip revealed mild osteoarthritis. Two months later lumbar spine magnetic resonance imaging (MRI) revealed degenerative disc disease, posterior disc bulging and facet arthropathy at L5-S1 resulting in moderate left foraminal stenosis. Subsequently, she was treated with physical therapy which included exercises, trans-cutaneous electrical nerve stimulation (TENS), lumbar traction and anti-inflammatory medications. Due to persistent symptoms, she followed-up with a neurosurgeon in January 2003. He ordered a lumbar spine CT scan which revealed mild spondylosis at L3-S1. The neurosurgeon diagnosed her with left L5 radiculopathy. She continued with physical therapy and anti-inflammatory medications and received three epidural injections which provided mild intermittent relief. Her symptoms persisted and in May 2003 she underwent elective left L5-S1 hemilaminectomy with diskectomy. She did not improve after the surgery and developed increased pain in her left lateral leg. A second MRI June 2003 revealed a large fluid collection that extended from the spinal canal through the laminectomy defect and into the subcutaneous tissue. This was thought to be a CSF fistula. She underwent a second surgery three weeks after the first surgery to repair a cerebral spinal fluid leak and recurrent extruded disk at L5-S1. She improved somewhat after the second surgery and was referred for a physical therapy strengthening program. Her symptoms however, persisted and she was unable to work.

The patient presented for chiropractic evaluation and treatment two and a half months after her second surgery. Her complaints were left lateral leg pain, described as a deep bone ache as if her leg was on fire. She stated her pain felt like a screwdriver being driven into her left lateral ankle. Her pain was rated as a constant but of variable intensity, level 2-8 out of 10 with an average pain of 4. She also presented with a new onset of right lumbosacral junction pain described as an ache rated as level 3 out of 10. She stated her low back pain was provoked when she leaned forward. She denied weakness, paresthesias, bowel or bladder loss or retention or thigh pain. Her left lower extremity complaint was aggravated with inversion of her left foot, internal rotation of her left hip, sitting, lying down and sleeping. Her symptoms were mildly relieved with walking, eversion of her left foot and if she performed a “figure four” of her left thigh.

Physical examination revealed an ectomorph body type standing 174 centimeters (68.5 inches) and weighing 57 kilograms (125 pounds). During the interview she sat the entire time with her right leg crossed over her left with her right foot wrapped behind her left ankle. Postural exam revealed decreased lumbar lordosis and thoracic kyphosis. Palpation reproduced tenderness over the left lateral leg inferior to the head of the fibula across the peroneal muscles. There was considered to be paraspinal tenderness and spasm at L2-L5 levels and over the posterior superior iliac spines, worse on the right side. Lumbosacral ranges of motion appeared normal. Deep tendon reflexes were 2+ patellas and 2+ achilles bilaterally. Plantar reflexes were down going. Hypesthesia was found over the left lateral leg and dorsum of her left foot. Motor examination found grade 4 weakness with dorsiflexion of the left foot and toes and eversion of the left foot. The remainder of the lower extremities muscles were graded 5/5. Her gait was normal. Straight leg raise was negative. Subjective tightness was found with the gastrocnemius and hip external rotator muscles. Passive inversion of the left foot reproduced leg pain.

She was instructed to stop crossing her legs and six days later she reported less pain along her left lateral leg and was sleeping better. She was followed for two months and discharged at that time symptom-free.

## Discussion

In the thigh, the peroneal division of the sciatic nerve supplies the short head of the biceps femoris muscle. Near the distal thigh, just above or in the popliteal fossa, the sciatic nerve divides into the tibial and common peroneal nerve (CPN). The CPN extends around the neck of the fibula where it is superficial and susceptible to direct trauma [14]. At the level of the fibular neck, the nerve passes beneath the peroneus longus tendon to enter the peroneal tunnel [15]. The CPN gives off the lateral cutaneous nerve of the calf proximal to the head of the fibula, which supplies sensation to the upper third of the anterolateral leg. After entering the lateral leg compartment deep to the peroneus longus tendon, the CPN divides into deep (DPN) and superficial peroneal (SPN) branches [16]. The DPN innervates the anterior compartment muscles, including tibialis anterior and extensor digitorum brevis, extensor hallucis, peroneus tertius, and extensor digitorum longus in addition to supplying the sensory branch between the first and second toes. Conversely, the SPN innervates the lateral compartment muscles of the leg including the peroneus longus and brevis muscles and supplies sensation to the lower 2/3 of the anterolateral leg and dorsum of the foot [17-19]. The CPN is most vulnerable as it becomes superficial over the fibular neck just distal to the head of the fibula [20], whereas the DPN and SPN are more vulnerable distally in the leg, ankle and foot [21]. Plantar flexion and inversion motions of the foot can stretch or compress the CPN in the peroneal tunnel [15,22].

Injury to the common peroneal nerve results in a foot drop described as slapping or tripping [20]. Pain may occur at the site of compression as well as distally into the lateral leg. At times there may be a radiation of pain into the thigh [15]. Numbness and tingling can occur along the lateral leg and dorsum of the foot [14]. Peroneal neuropathies occurring at the fibular neck affect the DPN more commonly than the SPN. Also with common peroneal neuropathies, weakness can be more prominent in muscles supplied by the DPN [22]. Neuropathy of both the DPN and the SPN will cause weakness with dorsiflexion of the foot and toes and eversion of the foot. If only the DPN is affected, there will be weakness with foot and toe dorsiflexion and sensory deficit to the web of skin between the first and second toes [22]. In severe cases of peroneal neuropathy there will be obvious foot drop. Milder cases of foot dorsiflexion weakness are assessed with heel walking and manual muscle testing. Sensory loss is typically found over the lateral leg and dorsum of the foot sparing the fifth toe [14]. Palpation or pressure over the peroneal tunnel region can reproduce the patient symptoms. Resisted inversion of the foot can also reproduce pain [15].

The history and exam may be the most helpful at arriving at a diagnosis for suspected peroneal neuropathy. Neurodiagnostic testing (EMG/NCV) may be required to provide a more complete understanding and can allow for localization of the lesion. Misidentification of the lesion can result in unnecessary surgery or delay in surgery. Electrodiagnostic testing can also establish that a physiologically relevant nerve injury is present and may also provide insight into the timing of injury and underlying pathophysiology of injury (demylination vs. axonal disruption). EMG should be used if the patient does not improve with time or treatment, is considered a surgical candidate secondary due to intractable pain or the presence of progressive weakness, or there are equivocal MRI findings [23].

MRI has utility in the evaluation for peroneal neuropathy as it can detect the proximal portion of the CPN at the knee for space occupying lesions, edema and change in nerve size. However, in most cases of peroneal neuropathy these findings are not evident. [21]. High-resolution Sonography may be used to detect structural lesions such as an intramural ganglion or inflammatory changes and color duplex ultrasonography and angiography may be used for assessment of vascular compromise including popliteal pseudo-aneurysm of the popliteal artery [24].

Clinical findings can aid in determining the etiology of a patient’s condition. L5 radiculopathy and peroneal neuropathy can both present with weakness of the foot dorsiflexors and toe extensors, however, L5 radiculopathy may present with weakness during foot inversion versus weakness with foot eversion associated with peroneal neuropathy [14]. Additionally, reflex changes at the patella, medial hamstring and Achilles tendon can distinguish a L4, L5 or S1 radiculopathy from a common peroneal neuropathy [25]. Sensory changes to light touch or pinprick may not improve the clinical picture as dermatomal patterns and peripheral nerve distributions can have much overlap and sensory evaluation may be prone to subjective bias [26]. Finally, adverse nerve root tension, including femoral nerve stress test and straight leg raise can indicate a lumbar nerve root involvement which is absent during peroneal neuropathy. On the other hand, passive or forceful ankle inversion tensions the peroneal nerve which may reproduce symptoms of a peroneal neuropathy [15].

Injury to the sciatic nerve, especially when the peroneal portion is affected, can mimic a common peroneal neuropathy at the fibular head. Partial sciatic nerve injuries usually affect the lateral division (common peroneal nerve) more commonly than the medial division (tibial nerve); this is believed to be due to limited supportive tissue surrounding the peroneal nerve and the fact the peroneal nerve is taut and secured at both its proximal and distal ends. These high sciatic nerve lesions can be caused by static notch injections, hip trauma, hip surgery and gluteal compartment hemorrhage. High sciatic nerve lesions are differentially diagnosed from common peroneal nerve lesions through needle EMG of the short head of the biceps femoris which receives innervation from the peroneal division of the sciatic nerve [27].

One must also consider the presence of other neuropathies, such as a diabetic neuropathy, which can present with foot drop and dysesthesias. Diabetic polyneuropathy generally occurs bilaterally versus unilaterally for a mononeuropathy or peripheral entrapment syndrome [11]. Diabetic symmetric distal polyneuropathy includes tingling, buzzing or a prickling sensation in a stocking distribution. Absent Achilles reflexes are a frequently encountered sign in diabetic polyneuropathy. Weakness generally involves the extensor hallucis longus muscles rather than dorsiflexion of the feet. Loss of vibration at the toes is also common with diabetic polyneuropathy [28]. Diabetic mononeuropathy or Mononeuritis multiplex can involve one nerve or multiple nerves. The cause is thought to be vasculitis, ischemia or infarction of the nerve. The onset is acute and self-limiting, generally resolving over six weeks. Entrapment syndromes begin slowly and continually progress until intervention [29]. Other conditions can produce peripheral neuropathies including human immuno-deficiency virus (HIV), nutritional deficits, polyarteritis nodosa, sarcoidosis, SLE, toxemia and uremia [14]. The patient in our case study did not exhibit signs or symptoms of a systemic condition.

## Conclusion

This case represents a patient who underwent L5-S1 hemilaminectomy and discectomy for the diagnosis of a L5 radiculopathy. After two surgeries and over a year of physical therapy the patient presented to the authors office. In this instance the authors had the foresight of the failed low back surgery leading to the diagnosis of a peroneal neuropathy. The importance of this case study is the differential diagnosis between a L5 radiculopathy and peroneal neuropathy. The patient’s sensory complaints, exam findings and imaging studies mimicked a L5 radiculopathy. There are indications however, pointing to a peroneal neuropathy, which included weakness with foot eversion along with dorsiflexion and toe extension, tenderness over the fibular neck and peroneal tunnel and a history of increased pain with ankle inversion and relief upon ankle eversion. Ideally, electrodiagnostic testing would have been performed prior to the second surgery and would have essentially ruled out lumbar spine involvement. Additionally, an EMG/NCV may have provided a more conclusive diagnosis of peroneal neuropathy, however, in this particular case, following a trial of treatment targeting the common peroneal nerve, the patient reported resolution of her symptoms thus eliminating the need for further diagnostic testing or clarification.

We presented a case of undiagnosed peroneal neuropathy in a female who presented to a chiropractic office that was non-responsive to two lumbar decompression surgeries and interventional pain procedures that responded to a simple direction to not sit cross legged. This adds to the existing literature of previously-reported cases and concludes that clinicians managing such patients must exhibit careful diagnostic acumen and clinical decision making to ensure proper patient treatment and management.

## Consent

Written informed consent was obtained from the patient for publication of this case report and any accompanying images. A copy of the written consent is available for review by the Editor-in-Chief of this journal.

## Competing interests

The authors declare that they have no competing interests.

## Authors’ contributions

Both authors wrote, reviewed and made editorial changes in the manuscript. Both authors read and approved the final manuscript.
